# Clinical Recovery Without Recanalization in Iron Deficiency Anemia-Associated Cerebral Venous Thrombosis: A Case Report

**DOI:** 10.7759/cureus.110689

**Published:** 2026-06-11

**Authors:** Shunichiro Hoshi, Takuya Moriwaki, Hidetoshi Mochida

**Affiliations:** 1 Neurosurgery, Asahi General Hospital, Asahi, JPN

**Keywords:** apixaban, cerebral venous thrombosis, ferritin, heavy menstrual bleeding, hemiparesis, iron deficiency anemia, mr venography, transverse sinus thrombosis, venous edema

## Abstract

Cerebral venous thrombosis (CVT) is an uncommon cerebrovascular condition with highly variable clinical presentations and a wide spectrum of underlying causes. Iron deficiency anemia (IDA) has been increasingly recognized as a significant predisposing factor for CVT. We describe the case of a 45-year-old woman who presented with a gradually intensifying headache, followed by acute onset of left facial palsy and left-sided weakness. Laboratory tests demonstrated microcytic, hypochromic anemia, consistent with IDA. Computed tomography (CT), magnetic resonance imaging (MRI), and magnetic resonance venography (MRV) revealed thrombosis of the right transverse sinus, accompanied by venous infarction and intracranial hemorrhage. An extensive workup showed no evidence of hereditary thrombophilia or autoimmune disease. Subsequent evaluation identified a uterine fibroid causing chronic menorrhagia, which was considered the primary source of her IDA. Anticoagulation therapy was started with low-molecular-weight heparin (LMWH) and later transitioned to apixaban. She also received iron supplementation and blood transfusion. Her neurological deficits gradually improved, with no progression of the intracranial hemorrhage and no new thrombotic events. Follow-up imaging showed substantial resolution of venous edema and mass effect; however, MRV continued to demonstrate persistent occlusion of the right transverse sinus. Anticoagulation was stopped four months after disease onset, and no recurrence occurred during subsequent follow-up. This case underscores several key clinical considerations. First, IDA resulting from chronic gynecologic blood loss should be recognized as a modifiable risk factor for CVT. Second, anticoagulation can be used safely in patients with hemorrhagic CVT, even in the presence of severe anemia. Third, good clinical and radiologic outcomes are possible despite incomplete venous recanalization. Collectively, these observations suggest that restoration of overall cerebral venous hemodynamics, rather than recanalization by itself, may be crucial for recovery from CVT.

## Introduction

Cerebral venous thrombosis (CVT) is a condition defined by the development of thrombi within the dural venous sinuses, cerebral veins, or both [1]. Its annual incidence is estimated at roughly three to four cases per million individuals, with women accounting for approximately 75% of adult cases [2].

A range of risk factors has been linked to CVT, such as the use of oral contraceptives, pregnancy or the postpartum period, thrombophilic disorders, and anemia. Among these, iron deficiency anemia (IDA) is recognized as a major predisposing factor, particularly in women, and is frequently attributable to chronic gynecologic blood loss from uterine fibroids or adenomyosis [3].

Standard management of CVT consists of anticoagulant therapy in conjunction with correction of the underlying triggering conditions. Anticoagulation is advised even when intracranial hemorrhage or anemia is present [4]. In contrast, the association between venous recanalization and clinical prognosis remains uncertain, and some patients exhibit only modest clinical recovery despite radiologic confirmation of complete recanalization [5].

In this report, we present a case of CVT related to IDA secondary to menorrhagia caused by uterine fibroids. Despite concurrent intracranial hemorrhage and severe anemia, the patient experienced favorable clinical recovery after receiving anticoagulation and anemia correction, even though venous sinus occlusion persisted. Using this case, we explore the clinical relevance of recanalization and the compensatory adaptations within the cerebral venous circulation in CVT.

## Case presentation

A 45-year-old woman with a premorbid modified Rankin Scale score of 0 presented with several days of progressively worsening head heaviness, followed by the sudden onset of left-sided facial paralysis and left hemiparesis. Her gynecologic history was notable for two pregnancies and two deliveries. She had never used hormonal contraceptives and had no prior thrombotic events.

On arrival, her vital signs were within normal limits: body temperature, 36.6°C; heart rate, 74 beats/minute; respiratory rate, 20 breaths/minute; oxygen saturation, 99% on room air; and blood pressure, 118/76 mmHg. Neurological examination demonstrated left facial palsy and mild weakness of the left upper and lower limbs, graded 4/5 on manual muscle testing. Sensation was intact, but she had mild dysarthria. There was no ocular deviation. Both pupils were 3.5 mm in diameter and reactive to light. Her Glasgow Coma Scale score was 15 (E4V5M6). Initial laboratory evaluation revealed IDA, with a hemoglobin level of 8.1 g/dL, hematocrit of 27.0%, mean corpuscular volume of 71.4 fL, mean corpuscular hemoglobin of 21.4 pg, and mean corpuscular hemoglobin concentration of 30.0 g/dL. Platelet and white blood cell counts were within the normal range at 249 × 10³/μL and 8.4 × 10³/μL, respectively. Iron studies showed a serum iron of 16 μg/dL, an unsaturated iron-binding capacity of 322 μg/dL, and a ferritin level of 28 ng/mL. Coagulation parameters were as follows: prothrombin time (PT), 11.6 seconds; PT-international normalized (INR) ratio, 1.02; activated partial thromboplastin time, 27.9 seconds; and D-dimer, 2.5 μg/mL (Table 1). C-reactive protein was elevated at 13.96 mg/dL, although there were no clinical features suggestive of systemic infection.

**Table 1:** Summary of major laboratory findings MCV: mean corpuscular volume, UIBC: unsaturated iron-binding capacity

Parameter	Patient Value	Reference Range	Interpretation
Hemoglobin	8.1 g/dL	12.0–16.0 g/dL	Severe anemia
Hematocrit	27.0 %	36–46 %	Low
MCV	71.4 fL	80–100 fL	Microcytic
Platelet	24.9 ×10⁴/μL	15–40 ×10⁴/μL	Normal
D-dimer	2.5 μg/mL	<0.5 μg/mL	Elevated
Ferritin	28 ng/mL	30–150 ng/mL	Low
Serum Iron	16 μg/dL	60–170 μg/dL	Low
UIBC	322 μg/dL	150–300 μg/dL	Elevated

An extensive workup for thrombophilia and autoimmune disorders, including protein C and protein S levels, antiphospholipid antibodies, antineutrophil cytoplasmic antibodies, and antinuclear antibodies, yielded results within normal limits, and no relevant abnormalities were identified. Homocysteine and thrombin-antithrombin III complex levels were also normal. Given the lack of other prothrombotic factors, the CVT was deemed most likely secondary to IDA of initially uncertain cause. 

Non-contrast computed tomography (CT) obtained on the day of admission showed a hyperdense lesion in the right transverse sinus, as well as a hypodense area and a small acute hemorrhage in the right temporal lobe (Figure 1). Magnetic resonance imaging (MRI) with fluid-attenuated inversion recovery (FLAIR) sequences demonstrated diffuse venous edema and obliteration of the cortical sulci in the right temporoparietal region (Figure 2). Magnetic resonance venography (MRV) revealed a complete lack of blood flow within the right transverse sinus (Figure 3). Digital subtraction angiography (DSA) demonstrated non-visualization of the right transverse sinus and congestion of the cortical veins (Figure 4). Based on these findings, a diagnosis of cerebral venous thrombosis involving the right transverse sinus and cortical veins was established.

**Figure 1:**
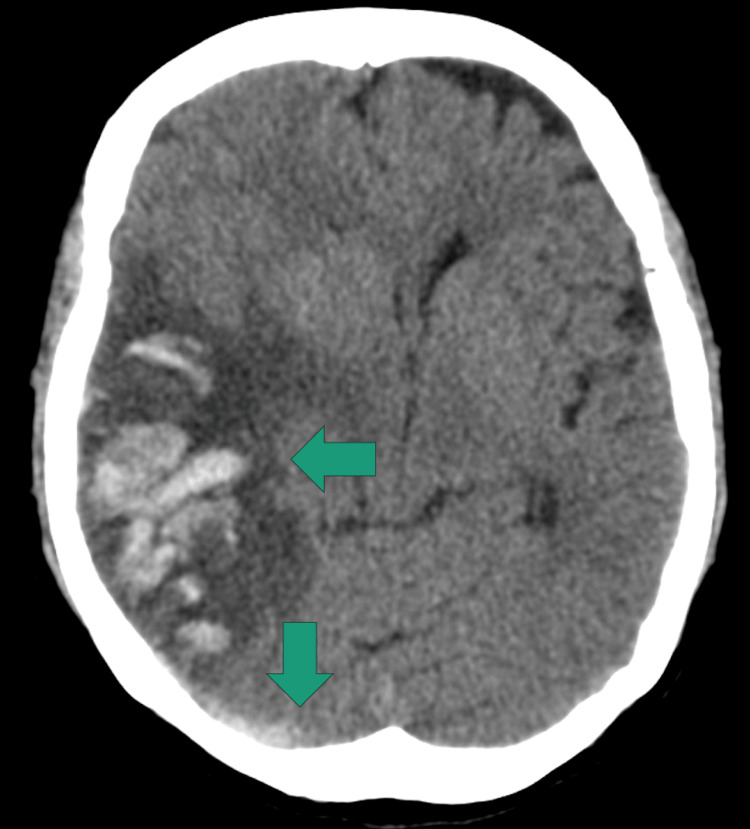
Non-contrast head CT on admission demonstrates hemorrhagic infarction with midline shift, along with a hyperdense lesion in the right transverse sinus. CT: computed tomography

**Figure 2:**
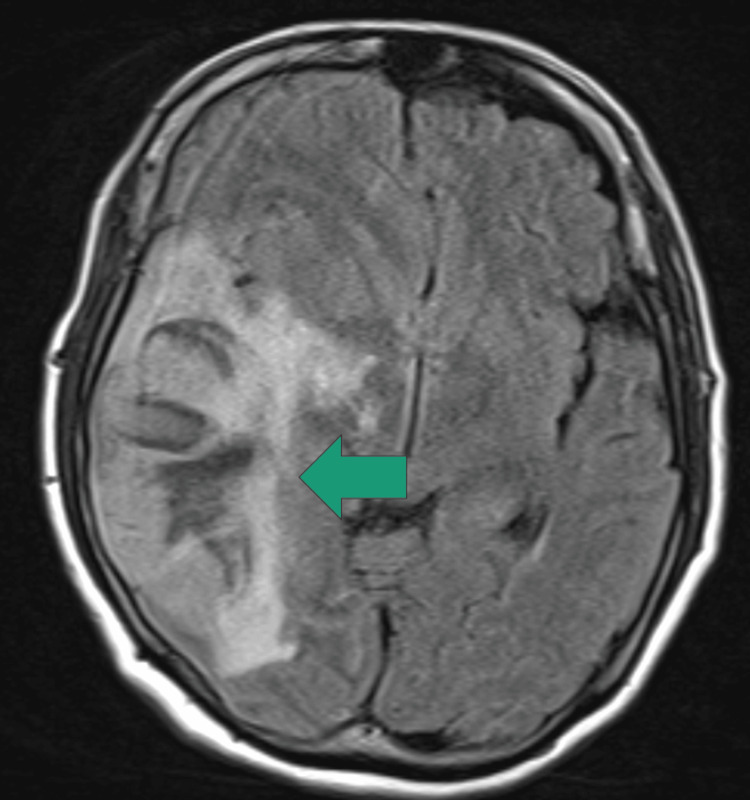
Axial view of MRI FLAIR sequence on admission demonstrates extensive edematous changes. MRI: magnetic resonance imaging; FLAIR: fluid-attenuated inversion recovery

**Figure 3:**
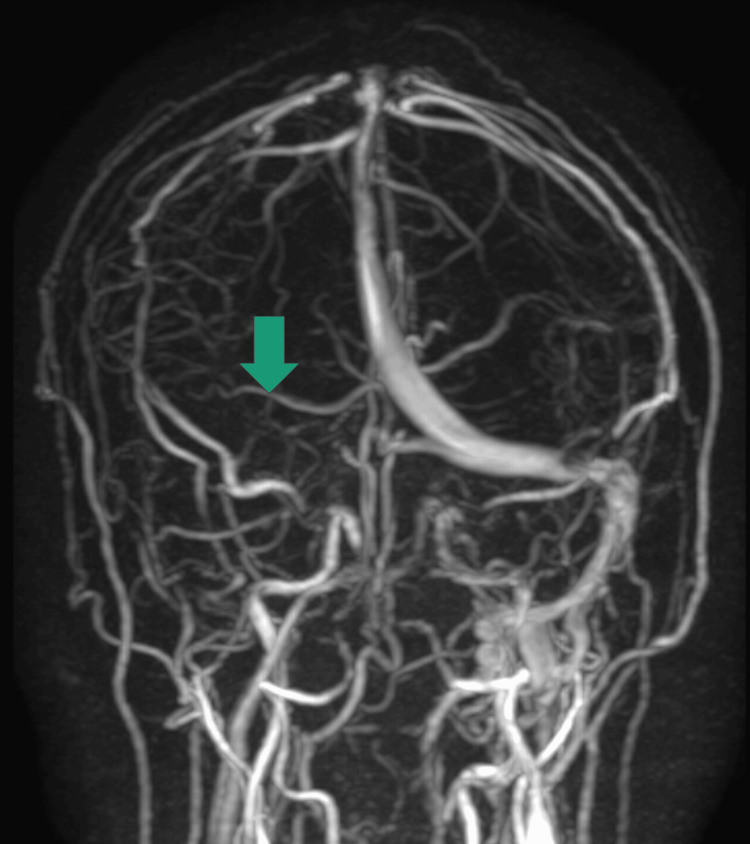
MRV shows absence of flow in the right transverse sinus. MRV: magnetic resonance venography

**Figure 4:**
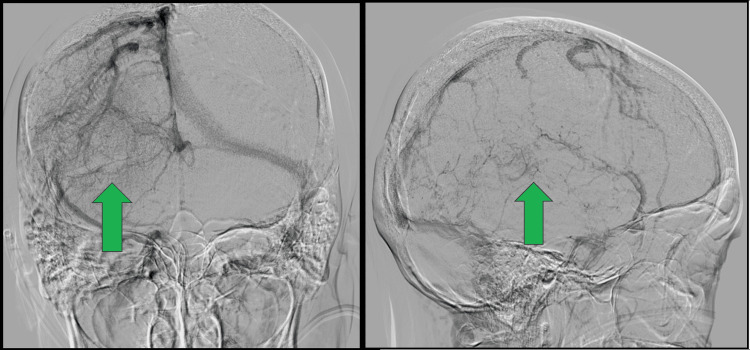
DSA performed on admission demonstrated non-visualization of the right transverse sinus and congestion of the cortical veins DSA: digital subtraction angiography

As part of the extended workup for IDA, a contrast-enhanced CT scan of the trunk was performed and revealed the presence of a uterine fibroid, with no evidence of underlying malignancy. Subsequent, more detailed history-taking uncovered a long-standing pattern of menorrhagia, indicating that chronic blood loss due to the fibroid was the most probable cause of IDA.

Anticoagulation was started immediately on day 0 with low-molecular-weight heparin (LMWH), together with iron supplementation and levetiracetam. Although endovascular thrombectomy was considered a potential treatment option, the patient's level of consciousness, neurological deficits, and radiological findings improved following initiation of anticoagulation therapy. Therefore, endovascular intervention was not performed, and conservative management was continued. By day 4, her hemoglobin had fallen to 6.8 g/dL, prompting transfusion of two units of red blood cells. After this, there was no additional decline in hemoglobin and no radiological or clinical evidence of worsening intracranial hemorrhage, and her neurological deficits gradually improved. Because her condition remained stable, anticoagulation was switched on day 14 to apixaban (2.5 mg twice daily). She continued to do well after the change in therapy and was discharged on day 21 with a modified Rankin Scale score of 1.

During outpatient follow-up, both the anemia and coagulation parameters showed continuous improvement. A follow-up non-contrast CT scan demonstrated reduction of cerebral edema and resolution of the midline shift (Figure 5). Follow-up MRI with FLAIR sequences showed substantial improvement in venous edema (Figure 6). In contrast, MRV still showed persistent occlusion of the right transverse sinus; nevertheless, the mass effect and midline shift had resolved (Figure 7).

**Figure 5:**
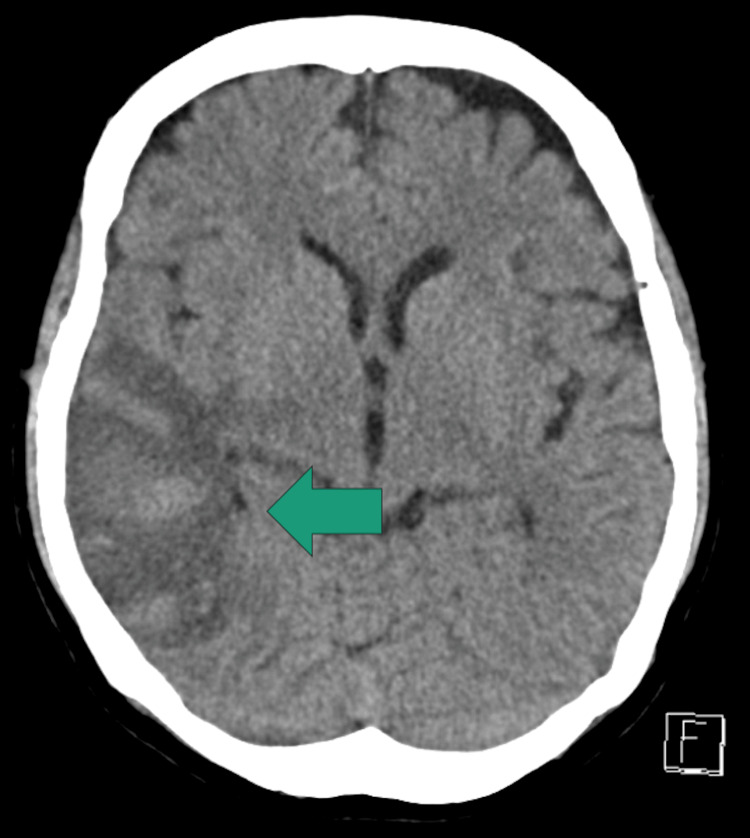
Non-contrast CT at three weeks after admission CT: computed tomography

**Figure 6:**
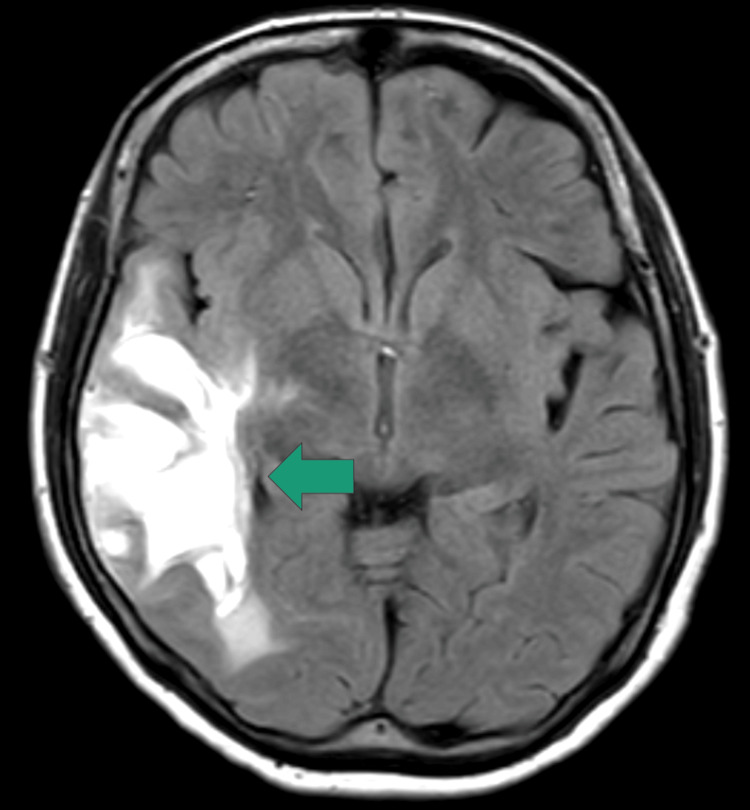
MRI FLAIR at three weeks after admission MRI: magnetic resonance imaging; FLAIR: fluid-attenuated inversion recovery

**Figure 7:**
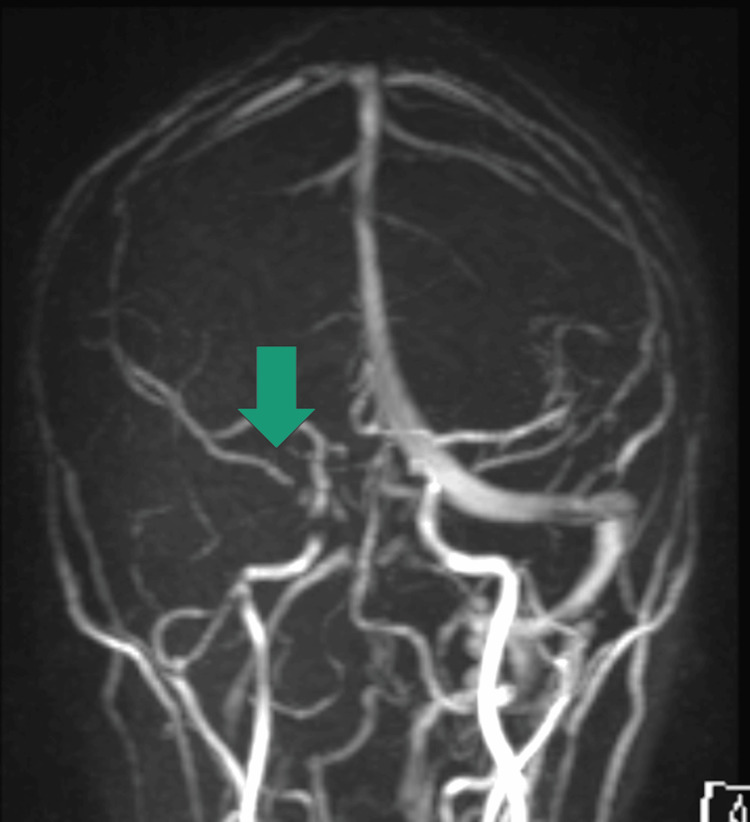
MRV at three weeks after admission MRV: magnetic resonance venography

Anticoagulation therapy was stopped after four months, and no recurrence occurred during the following 1.5-year observation period. A later gynecological assessment showed that the uterine fibroid, measuring 36 × 28 mm, remained stable in size. As menstrual bleeding did not increase, conservative management with oral iron supplementation and scheduled follow-up visits was maintained after thoroughly weighing both surgical and medical treatment options.

## Discussion

IDA is a well-established risk factor for CVT and is reported to be present in about 9-27% of all CVT cases [4]. The leading cause of IDA is chronic gynecologic blood loss, most frequently due to uterine fibroids or adenomyosis; nonetheless, gastrointestinal bleeding, malabsorption, inadequate dietary iron intake, and various environmental influences are also recognized etiologies. In numerous CVT cases associated with IDA, additional prothrombotic factors, such as oral contraceptive use or inherited/acquired thrombophilia, are identified; however, in roughly 30% of patients, IDA is the sole detectable risk factor [3]. In the current case, the patient had no history of oral contraceptive use and no laboratory evidence of thrombophilia, indicating that IDA secondary to menorrhagia from uterine fibroids was the main factor contributing to the onset of CVT. Several pathophysiological mechanisms have been suggested to account for the link between IDA and CVT, including impaired erythrocyte deformability, systemic endothelial activation, and reactive thrombocytosis [3]. In this patient, pronounced microcytic anemia was noted, which may have promoted thrombus formation.

Anticoagulation therapy continues to be the mainstay of CVT treatment. During the acute phase, management typically begins with unfractionated heparin or LMWH and is then transitioned to long-term therapy using vitamin K antagonists (VKAs) or direct oral anticoagulants (DOACs). Notably, the presence of intracranial hemorrhage is not regarded as a contraindication to anticoagulation [1,6,7], and anticoagulation is likewise advised for patients with IDA-related CVT. Recent evidence indicates that LMWH is often chosen as the initial therapeutic option for IDA-associated CVT [3]. In the case presented here, LMWH was started despite concurrent intracranial hemorrhage, and the patient experienced a good clinical outcome without any hemorrhagic complications.

In the context of long-term anticoagulant therapy, comparative studies of DOACs versus VKAs have shown no meaningful differences in the incidence of complete recanalization, recurrent venous thromboembolism (VTE), major bleeding, intracranial hemorrhage, or overall mortality. Apixaban, however, seems to be linked to a lower risk of recurrent VTE, major bleeding, intracranial hemorrhage, and death. While some evidence indicates that VKAs may produce higher rates of complete recanalization, it is still uncertain whether complete venous recanalization is required for clinical improvement [5]. In addition, low-dose apixaban (2.5 mg), prescribed for extended VTE prophylaxis, has been associated with both a decreased risk of bleeding and a lower rate of recurrence [8]. This makes it a potentially suitable choice for patients with a high bleeding risk, including those with intracranial hemorrhage or IDA due to menorrhagia, as in the case described. However, it should be noted that low-dose apixaban is not a standard treatment regimen for acute CVT, and its use in the present case reflected an individualized risk-benefit assessment based on the patient's elevated risk of bleeding.

Although endovascular treatment (EVT) may theoretically provide more rapid recanalization in patients with CVT, its impact on clinical outcomes remains uncertain [9]. The randomized clinical TO-ACT trial (Thrombolysis or Anticoagulation for CVT) failed to demonstrate a clinical benefit of EVT over standard anticoagulation therapy in patients with severe CVT [10]. Therefore, current evidence supports anticoagulation as the first-line treatment for CVT, while EVT is generally reserved as a rescue therapy for patients who experience clinical deterioration, fail anticoagulation therapy, or have contraindications to standard medical treatment [4]. In the present case, neurological deficits, level of consciousness, and radiological findings improved following initiation of LMWH, and no progression of intracranial hemorrhage was observed. Consequently, anticoagulation alone was considered sufficient, and endovascular intervention was not pursued. However, given the presence of transverse sinus occlusion and hemorrhagic venous infarction, EVT would have been considered if clinical or radiological deterioration had occurred despite adequate anticoagulation.

Management of IDA involves administering iron supplements either orally or intravenously, along with red blood cell transfusions when indicated. While there are no dedicated guidelines for treating anemia in patients with stroke or CVT, emerging data suggest that correcting anemia may contribute to improved clinical outcomes [11]. In this case, the patient’s anemia resolved with iron supplementation and blood transfusion, and no further episodes occurred. When IDA is secondary to gynecological bleeding, gynecologic procedures such as myomectomy or hormonal therapy may be necessary [3], underscoring the need to identify the underlying etiology and implement a multidisciplinary treatment approach.

A noteworthy aspect of this case was the improvement in clinical symptoms and the resolution of venous edema despite persistent occlusion of the right transverse sinus. One possible explanation is the development of compensatory collateral venous drainage, although collateral pathways were not directly demonstrated in this case. Functional recovery in CVT is therefore not strictly contingent upon complete radiological recanalization [5]. In addition to recanalization status, adaptive changes in venous drainage and other factors may contribute to favorable clinical outcomes. Furthermore, correction of IDA may have helped prevent further thrombus propagation and improve cerebral venous hemodynamics. Collectively, these findings highlight the importance of addressing the underlying condition alongside anticoagulation in the comprehensive management of CVT.

Although the prognosis of CVT is generally good, a subset of patients still experience severe disability or death. Adverse prognostic factors include intracranial hemorrhage, deep venous system involvement, impaired consciousness, and underlying malignancy [12-14]. More recently, anemia at presentation has also been identified as an independent predictor of unfavorable functional outcome [14,15]. In the present case, despite intracranial hemorrhage and marked IDA, the patient achieved a good outcome owing to early diagnosis, appropriate anticoagulation, correction of anemia, and treatment of the precipitating disorder. In summary, the therapeutic objectives in CVT should extend beyond simple recanalization of the obstructed venous sinus. Comprehensive management, including anticoagulation and correction of underlying conditions such as IDA, is essential for optimizing clinical outcomes.

## Conclusions

IDA should be regarded as a significant risk factor for CVT, especially in women experiencing menorrhagia. In this case, good clinical and radiological outcomes were obtained following adequate anticoagulation and correction of IDA, even though clear recanalization of the affected venous sinuses was not demonstrated. This observation suggests that favorable recovery may occur despite persistent sinus occlusion in some patients with CVT. Possible explanations include adaptive changes in venous drainage and effective management of underlying conditions; however, the mechanisms underlying recovery could not be directly established in this case. Consequently, a holistic therapeutic approach that targets both the thrombotic event and its contributing factors may be crucial for achieving optimal results in patients with CVT.
